# Absence of Relevant Thermal Conversion of Cannabidiol to Tetrahydrocannabinol in E-Cigarette Vapor and Low-Tetrahydrocannabinol Cannabis Smoke

**DOI:** 10.1089/can.2022.0163

**Published:** 2022-10-12

**Authors:** Pascal Hindelang, Andreas Scharinger, Patricia Golombek, Miriam Laible, Sandra Tamosaite, Stephan G. Walch, Dirk W. Lachenmeier

**Affiliations:** ^1^ Chemisches und Veterinäruntersuchungsamt (CVUA) Karlsruhe, Karlsruhe, Germany.; ^2^ Chemisches und Veterinäruntersuchungsamt (CVUA) Sigmaringen, Sigmaringen, Germany.

**Keywords:** cannabidiol, tetrahydrocannabinol, hemp, *Cannabis sativa*, cannabis smoking, electronic cigarettes, risk assessment

## Abstract

**Introduction:**

Recent research claimed that CBD in commercial electronic cigarette (e-cigarette) liquids can be converted into psychotropic amounts of Δ^9^-THC. This study aims to validate this claim using a realistic e-cigarette setup. In addition, this study also investigates if such a conversion may occur during smoking of CBD-rich cannabis joints.

**Materials and Methods:**

Two different CBD liquids were vaporized using two different e-cigarette models, one of which was operated at extreme energy settings (0.2 Ω and 200 W). The smoke of six CBD joints was collected using a rotary smoking machine according to ISO 4387:2019. Analyses were conducted using nuclear magnetic resonance spectrometry as well as liquid chromatography tandem mass spectrometry.

**Results:**

For the condensed e-cigarette liquids, no increase in THC concentration could be observed. For the CBD joints, no THC formation was provable. The recovered THC concentrations were ranging between 1% and 48% (0.034 and 0.73 mg) of the THC amount initially contained in the joints before smoking.

**Conclusions:**

Using realistic conditions of consumer exposure, relevant conversion of CBD to THC appears to not be occurring. The health risk of CBD liquids for e-cigarettes, as well as low-THC cannabis intended for smoking, can be assessed by concentrations in the source material without the need to consider significant changes in psychotropic compounds during use by consumers.

## Introduction

E-liquids containing the nonpsychotropic CBD as well as CBD-rich but Δ^9^-THC-poor varieties of cannabis are offered for consumption, but depending on the jurisdiction are not allowed to exceed certain thresholds of THC.^1–3^ Unlike international standards for evaluating tobacco cigarettes, which typically apply routine analytical cigarette smoking machines, the regulatory acceptability of cannabis preparations for vaping or smoking is currently determined by analyzing the e-liquid or low-THC cannabis in the preparation as it is sold. Recent research has questioned this practice, as it has been claimed that CBD can be converted to THC by thermic vaporization of commercial e-liquids in commercial electronic cigarettes (e-cigarettes) in significant amounts (42–70% of decomposition products).^4^

The authors suggested to reconsider the viewpoint that CBD in e-cigarette liquids does not appear to have any psychotropic effect or any harmful effect on human health. However, a study by Kintz measured CBD concentrations in blood samples from consumers of CBD-containing e-liquids without Δ^9^-THC being detected in blood samples.^5^ Regarding smoking of low-THC cannabis, Gelmi et al observed blood THC concentrations at levels reported to cause impairment symptoms, but were unable to confirm such effects in a randomized double-blind placebo-controlled two-way crossover study.^6^ In a similar clinical trial, Arkell et al reported considerably lower blood THC concentrations and excluded clinically important impairment.^7^

To further investigate whether the conversion of CBD to Δ^9^-THC occurs in CBD e-cigarettes and low-THC cannabis smoke, and if future risk assessment would need to consider this effect, this study is the first to measure Δ^9^-THC concentrations in condensates from vaporized e-liquids and low-THC cannabis smoked using a routine analytical cigarette smoking machines.

## Materials and Methods

CBD e-liquids and low-THC cannabis were taken from samples from retail sale in Germany submitted to our institutes for regulatory control purposes.

The e-liquids contained CBD in concentrations of 55 g/L CBD and 100 g/L CBD in a propylene glycol/glycerol matrix. The liquids were loaded into an e-cigarette. The mouthpiece (“Drip Tip”) of the e-cigarette was connected to a syringe (volume 60 mL) through a flexible PVC tube. This study used two commercial e-cigarette devices. The first one (device 1) was eGo AIO All-in-One Style, 5–20 W, 1700 mAh, Vaporizer type BF SS316, 0.5 Ω (Shenzhen Joyetech Co., Ltd., Shenzhen, China).

As it became quickly evident that no THC had been formed with this device, a second device (device 2) allowing higher energy setting was purchased: Geekvape Ageis Legend Kit with additional vaporizer coils and Geekvape Z Sub-Ω Tank (Geekvape Z Series coils; Geekvape, Shenzen, China). The settings for the e-cigarette coils were set to a maximum of 0.2 Ω and 200 W to allow a maximum amount of heat for the vaporization process of the e-liquids. Before vaporization, each e-liquid was loaded into an individual refillable e-cigarette tank with a new vaporizer coil. The airflow control of the e-cigarette was adjusted to allow maximum air intake.

The vaporization was conducted stepwise until the syringe was filled with vapor to the 60 mL mark. The syringe was then removed from the PVC tube, sealed with multiple layers of parafilm, and placed on a laboratory bench until the vapor was condensed completely.

The condensate was collected by inserting 2 mL of deuterated methanol (MeOD) into the syringe. From the condensate methanol mixture, 600 μL were transferred to a nuclear magnetic resonance (NMR) tube for the quantification of Δ^9^-THC and CBD in a Bruker 400 MHz Ultrashield NMR spectrometer (Bruker, Rheinstetten, Germany). The NMR method was previously described in detail.^8^ As a reference sample, the same amount of unvaporized e-liquid was dissolved in 2 mL of MeOD and also prepared for measurement.

From each remaining sample solution, which has been obtained from vaporizing the liquids at 0.2 Ω and 200 W, dilutions were prepared for the quantification of Δ^9^-THC and CBD using a previously described liquid chromatography tandem mass spectrometry (LC-MS/MS) method^9^ with the following modifications to improve separation of cannabinoids: separation column Raptor, ARC-18, 2.7 μm, 150×2.1 mm (Shimadzu Deutschland GmbH, Duisburg, Germany). The separation was isocratic with 20% water and 80% methanol, containing 0.1% of formic acid.

For low-THC cannabis, joints were prepared using 1 g of cannabis per joint. Using a routine analytical cigarette smoking machine according to ISO 3308:2012 (RM 20 H; Borgwaldt, Hamburg, Germany) the joints were smoked without prior conditioning and the combined smoke of five joints adsorbed on a filter paper (92 mm). The filter paper was then extracted with methanol and the extract was analyzed using LC-MS/MS.^9^

Institutional review board approval was not required because this study was purely experimental and did not involve any human participants.

## Results

Table 1 presents the recovery of Δ^9^-THC in the condensates of vaporized liquids compared with the Δ^9^-THC concentrations measured in pure CBD liquids. The results indicate that for none of the settings investigated for the e-cigarette, Δ^9^-THC could be detected in the obtained condensates by using ^1^H NMR spectroscopy. LC-MS/MS measurements have confirmed that an increase in Δ^9^-THC concentration at the highest energy setting did not occur.

**Table 1.** Relative Increase in Δ^9^-Tetrahydrocannabinol Concentrations in Recovered Condensates After Vaporization of E-Liquids

**Power settings/device type**	**Liquid A (3 mg/L Δ^9^-THC^a^)**	**Liquid B (9 mg/L Δ^9^-THC^a^)**
0.5 Ω; 23 W/device 1	No increase in Δ^9^-THC detected^b^	No increase in Δ^9^-THC detected^b^
0.6 Ω; 28 W/device 1	No increase in Δ^9^-THC detected^b^	No increase in Δ^9^-THC detected^b^
0.2 Ω; 80 W/device 2	No increase in Δ^9^-THC detected^b^	No increase in Δ^9^-THC detected^b^
0.2 Ω; 120 W/device 2	No increase in Δ^9^-THC detected^b^	No increase in Δ^9^-THC detected^b^
0.2 Ω; 150 W/device 2	No increase in Δ^9^-THC detected^b^	No increase in Δ^9^-THC detected^b^
0.2 Ω; 200 W/device 2	No increase in Δ^9^-THC detected^b,c^	No increase in Δ^9^-THC detected^b,c^

^a^ Original liquids measured using LC-MS/MS.

^b^ Measurements were conducted using ^1^H NMR spectroscopy.

^c^ Condensates obtained at the highest power setting were additionally measured using LC-MS/MS.

LC-MS/MS, liquid chromatography tandem mass spectrometry; NMR, nuclear magnetic resonance.

The results of Δ^9^-THC recovered during the ISO smoking regime are shown in Table 2. About 1–48% of Δ^9^-THC (0.034–0.73 mg) initially contained in the cannabis plant material was found.

**Table 2.** Recovery of Δ^9^-THC After Smoking of Low THC Cannabis

**Sample**	**Δ^9^-THC before smoking (per 5 joints containing 1 g of cannabis)**	**Δ^9^-THC recovered during ISO 4387:2019 smoking regime**
1^a^	2.02 mg	0.200 mg (10%)
2^a^	1.26 mg	0.070 mg (6%)
3^b^	3.19 mg	0.034 mg (1%)
4^b^	3.30 mg	0.130 mg (4%)
5^b^	2.83 mg	0.240 mg (8%)
6^b^	1.52 mg	0.730 mg (48%)

^a^ Smoking regime strictly according to the ISO standard conditions (as for tobacco cigarettes), that is, one puff every 60 sec with a puff duration of 2 sec and 35 mL puff volume.

^b^ After the joints ran out between the puffs and had to be relit manually, the puff parameters for the other four samples were changed as follows: puff frequency 50 sec, puff duration 5 sec, and puff volume 55 mL. Smoking then worked much better.

## Discussion

^1^H NMR spectroscopy of Δ^9^-THC in condensates indicates that an overall formation of Δ^9^-THC does not occur in commercial CBD liquids. These findings are consistent with previous findings from Kintz.^5^ Further analytical measurements using more sensitive LC-MS/MS have confirmed that no formation of Δ^9^-THC after heating occurred. Note that a decrease in CBD concentrations in condensates has not been observed in any measured sample using NMR, therefore also confirming that thermic conversion of CBD to Δ^9^-THC did not occur.

Czégény et al reported a high conversion rate of CBD to Δ^9^-THC (corresponding to 0.5–1 mg/mL e-liquid).^4^ However, the study was conducted with pure CBD in methanol solution instead of an e-cigarette matrix (e.g., propylene glycol and glycerol) and without a realistic e-cigarette setup, since the measurements were purely conducted using a pyrolysis gas chromatography system (i.e., without any e-cigarette setup before analysis). Therefore, the authors do not believe that the results of Czégény et al^4^ have any practical value and do not allow one to make judgment about e-cigarettes. The authors also believe that the interpretation of Czégény et al^4^ to reconsider psychotropic effects of CBD liquids is not founded in the data of the study, and clearly must be rebutted by the data from this study.

Similarly, our results show that by smoking of low-THC cannabis, only a fraction of THC is recovered in the smoke. Although cannabis contains other potential THC precursors besides CBD, the formation of THC from CBD or other precursors can be excluded in this case as well. Even the case of the highest THC concentration recovered in our samples (0.73 mg/5 joints) would be below psychotropic levels.^10^ This is in line with other data showing that inhalation of vaporized and combusted CBD-dominant cannabis preparations did not result in any detectable cognitive impairment of study participants regarding driving behavior.^6,7^

Concerning consumer safety, it is not recommended to consume CBD liquids or e-liquids in general with the respective power outputs tested in this investigation (possibly in the false belief to produce psychotropic levels of THC). For example, harmful substances such as benzene can be formed from propylene glycol and glycerol, especially in high-power settings.^11^ Note that the e-cigarette itself heated up, so concerns regarding the safety and reliability of the device may arise when operated in the settings used.

Furthermore, the high temperature of the vaporized liquid and the heating of the mouthpiece during the vaporization procedure might also cause severe injuries to lung tissue and burns to the mouth and oral epithelium, respectively. It should also be noted that upon inhalation at the highest temperature settings, the condensate and the remaining CBD liquid had a strongly unpleasant ammonia smell, which would make the consumption of CBD liquids with the respective settings impossible. This was observed in both liquids and may result from matrix and aroma compounds or noninert components of the device (such as sealants and the vaporizer coil), which could have been broken down or disintegrated by high temperatures resulting from high power output.

Consumers should also be warned against vaping other unregulated compounds such as synthetic or semisynthetic cannabinoids. For example, in a very similar experimental setup to this study, Munger et al^12^ were recently able to show that vaping of Δ^8^-THC may form the toxic compound ketene that had been associated as underlying mechanism for the cause of e-cigarette, or vaping, product use associated lung injury (EVALI) as it may also be formed from vitamin E acetate, which had been contained in some CBD e-liquids.^12,13^ The consideration that EVALI was not caused by cannabinoids but other compounds in the e-cigarette liquids was recently validated by a risk assessment of inhaled CBD and THC, which found that the intake would be typically below thresholds of toxicity.^14^ Nevertheless, it was also remarked that the available data for risk assessment of CBD is rather limited. Caution is also advised because CBD products may potentially lead to positive urine drug tests due to significant contamination with THC and other cannabinoids.^15^

## Conclusions

In general, the vaporization or burning of natural materials such as cannabis extracts or cannabis plant materials is a multifactorial process that is characterized by various steps from acidic forms of cannabinoids to neutral cannabinoids and further oxidation products. Our results show that CBD-rich cannabis under vaporization or burning conditions does not form Δ^9^-THC concentrations. The authors, therefore, believe that the risk of consumer products based on low-THC cannabis or hemp can be further assessed for regulatory purposes by direct analysis of the products as such.

## Abbreviations Used

CBD: cannabidiol
e-cigarette: electronic cigarette
EVALI: e-cigarette, or vaping, product use associated lung injury
LC-MS/MS: liquid chromatography tandem mass spectrometry
MeOD: deuterated methanol
NMR: nuclear magnetic resonance
THC: tetrahydrocannabinol
